# Reviews

**Published:** 1883-01

**Authors:** 


### Reviews.

**Microscopical Morphology of the Animal Body in Health and Disease.** By C. Heitzmann, M. D., late Lecturer on Morbid Anatomy in the University in Vienna, Austria. With 380 original engravings. New York: J. H. Vail & Co., 21 Astor Place. 1883.

We have here a very valuable book, embodying the results of ten years' intense labor by one admirably fitted for the task to which he had devoted himself. The volume also records the labors of several men who have devoted much time to original investigation under Dr. Heitzmann's direction. Viewed as a whole, the work will add fresh laurels to American authorship. We think, however, that the author claims more for the microscope than will ever be realized by that already invaluable instrument. He claims to be able to tell the constitution of a person, without knowing anything of his former life, by a microscopical examination of his blood, and proposes that marriages should be permitted in doubtful cases only upon the permit of a reliable microscopist. As an illustration, he gives the following incident, which sounds rather Salisbury-like: A young physician fell in love with his cousin, and the cousin reciprocated the attachment; but the physician's *science* prevented him from falling head and ears over in love so as to be blind to consequences, and Dr. H. was, therefore, consulted. The result we give in his own words: "I examined his blood and told him that he was a 'nervous' man, passing sleepless nights, and had a moderately good constitution. The condition being suspected in the kindred lady, marriage was not advisable, for fear that the offspring might degenerate. So great was his faith in my assertion that he gave up the idea of marrying his cousin, offering her the last chance, viz., the examination of her blood. This beautiful girl came to my laboratory, and very much to my surprise I found, upon examination of her blood, a first-class constitution. The next day I told the gentleman: 'You had better marry her.'" We do not give this interesting little story as a sample of the book, by any means. It is impossible, in the limited space at our command, to call attention to its really admirable qualities. The nineteenth and twentieth chapters, which treat of the "Urinary Tract" and "The Urine," are alone well worth the price of the book to any scientific physician. Again, we repeat, the book is an admirable, and, in many respects, an original one, and no physician's library is complete without it. The illustrations are all made directly from Dr. Heitzmann's drawings; and it is refreshing to see a book with original plates. The publishers have brought out the book in excellent style. The print is clear and distinct, and the volume contains nearly 900 pages.

---

**A System of Surgery, Pathological, Diagnostic, Therapeutic and Operative.** By Samuel D. Gross, M. D., LL. D., D. C. L., Oxon., LL. D., Cantab., Emeritus Professor of Surgery in Jefferson Medical College. Illustrated by upwards of 1600 engravings. Sixth edition; thoroughly revised and greatly improved; in two volumes. Philadelphia: H. C. Lea's Son & Co. 1882.

Already some years have passed since the whole profession united in testifying their appreciation of the noble life and work of "America's most distinguished surgeon" on the occasion of the semi-centennial of his connection with medicine. Few, we think, supposed that he would add new honors to his name, after having so well served the profession and mankind for more than three score years and ten. It is but recently that we noted, with regret, that he had laid down the crown of the teacher; but it is with surprise and delight that we find that he has taken up anew the sceptre of the author. The first edition of "Gross' Surgery" appeared in 1859. From time to time, as the editions were exhausted, it was revised and improved; but, we doubt if any of the previous revisions evidence so much labor, thoroughness in revision and general improvement, as does this sixth edition. Almost every chapter has been re-written, a large amount of new matter introduced, and the whole work is a complete and faithful exponent of the art and science of surgery, as it exists to-day. To Prof. Lister he accords high praise as one of the world's eminent benefactors. He has the wisdom, too, to appreciate the triumphs accomplished by specialists, and has called in some of the most distinguished of them to aid him in the preparation of the chapters on the respiratory organs, the eye and ear. E. C. Sequin contributes a lecture on cranio-cerebral topography, and Batty and Jayre have respectively contributed valuable matter relative to Oöphorectomy, and the treatment of spinal diseases. The general plan of the work is the same as in previous editions, which has met with general favor. H. C. Lea's Son & Co. have done their part of the work in a way worthy of themselves and of the book.

